# Dissecting the sequential evolution of a selfish mitochondrial genome in *Caenorhabditis elegans*

**DOI:** 10.1038/s41437-024-00704-2

**Published:** 2024-07-05

**Authors:** Joseph J. Dubie, Vaishali Katju, Ulfar Bergthorsson

**Affiliations:** 1 Department of Veterinary Integrative Biosciences, Texas A&M University, College Station, TX USA; 2 Department of Integrative Biology, University of Texas, Austin, TX USA; 3 Evolutionary Biology, Department of Ecology and Genetics, Uppsala University, Norbyvägen 18D, 752 36 Uppsala, Sweden

**Keywords:** Molecular evolution, Experimental evolution

## Abstract

Mitochondrial genomes exist in a nested hierarchy of populations where mitochondrial variants are subject to genetic drift and selection at each level of organization, sometimes engendering conflict between different levels of selection, and between the nuclear and mitochondrial genomes. Deletion mutants in the *Caenorhabditis elegans* mitochondrial genome can reach high intracellular frequencies despite strongly detrimental effects on fitness. During a mutation accumulation (MA) experiment in *C. elegans*, a 499 bp deletion in *ctb-1* rose to 90% frequency within cells while significantly reducing fitness. During the experiment, the deletion-bearing mtDNA acquired three additional mutations in *nd5*, namely two single insertion frameshift mutations in a homopolymeric run, and a base substitution. Despite an additional fitness cost of these secondary mutations, all deletion-bearing molecules contained the *nd5* mutations at the termination of the MA experiment. The presence of mutant mtDNA was associated with increased mtDNA copy-number. Variation in mtDNA copy-number was greater in the MA lines than in a wildtype nuclear background, including a severe reduction in copy-number at one generational timepoint. Evolutionary replay experiments using different generations of the MA experiment as starting points suggests that two of the secondary mutations contribute to the proliferation of the original *ctb-1* deletion by unknown mechanisms.

## Introduction

Mitochondria are essential components of nearly all eukaryotic cells, having originated through the endosymbiosis of an ancient prokaryote in an archaebacterium (Martin and Müller 1998) or basal eukaryote (Margulis 1970; Doolittle 1980). While the two main competing hypotheses regarding mitochondrial origin differ with respect to the nature of the host (Martin et al. 2001; Koonin 2010; Gray 2012), many of the cellular processes of this ancient prokaryote have been taken over by its host during more than one billion years of evolution. As such, mitochondria have greatly reduced genomes and primarily exist to produce energy for their host cells (Lang et al. 1997). Within the animal kingdom, with few exceptions, mitochondrial genomes contain the same limited set of components: 13 proteins, two rRNA, and 22 tRNA encoding regions (Kairo et al. 1994). This drastic reduction of the mitochondrial genome dictates its reliance on the nuclear gene products of eukaryotic cells. The eukaryotic nuclear genome not only controls functions like translation, transcription and replication for mitochondria (Burton et al. 2013), but also produces the majority of proteins that interact with mtDNA-encoded proteins to create the complexes involved in the electron transport chain (ETC). This mitonuclear interaction has resulted in tight coevolution between mitochondrial and nuclear-encoded subunits and is reflected in cases of “mitonuclear mismatch” where hybrids with incompatible nuclear and mitochondrial genomes show reduced fitness and ETC efficiency (Pinto and Moraes 2015).

The population dynamics of mitochondrial genes differ in notable aspects from that of nuclear genes which, in turn, can facilitate an increase in the frequency of deleterious mitochondrial variants. First, the predominantly uniparental inheritance of mitochondria can result in cytonuclear conflict (Cosmides and Tooby 1981; Havird et al. 2019). Classic examples of cytonuclear conflict include cytoplasmic male sterility in flowering plants and Mother’s curse (Gemmell et al. 2004; Fujii et al. 2011). Second, a single cell contains multiple copies of the mitochondrial chromosome, thereby engendering competition between different mitochondrial genotypes (mitotypes) (Eberhard 1980; Cosmides and Tooby 1981). Deleterious mitochondrial DNA mutations can increase in frequency by assuming some form of transmission or replicative advantage (Eberhard 1980; Clark et al. 2012; Havird et al. 2019; Dubie et al. 2020; Sequeira et al. 2024). The biased transmission of deleterious mutations is the hallmark of selfish DNA and the mechanisms that favor them in mtDNA are poorly understood (Havird et al. 2019). Some of the best-studied examples of selfish mtDNA mutations are deletions in yeast, *Drosophila melanogaster* and *Caenorhabditis* nematodes (MacAlpine et al. 2001; Taylor et al. 2002; Tsang and Lemire 2002; Clark et al. 2012; Lin et al. 2016; Ma and O’Farrell 2016; Dubie et al. 2020; Sequeira et al. 2024). The simplest explanation for a replicative advantage of deleterious mtDNA deletions is that smaller mtDNA molecules can replicate faster than a full-length mtDNA molecule, referred to as the “small genome hypothesis” (Wallace 1992). However, mtDNA base substitutions and small deletions can sometimes expand clonally at a rate that is similar to large mtDNA deletions (Campbell et al. 2014). Furthermore, experiments in *C. elegans* have failed to find support for the “small genome hypothesis” (Gitschlag et al. 2016). In yeast mtDNA petite mutants, an expansion of replication origins appears to play a large role for their replicative advantage (MacAlpine et al. 2001). Experiments in *Drosophila* and *Caenorhabditis* suggest that an increase in mtDNA copy-number and changes in mitochondrial fusion-fission cycles can favor mtDNA mutants (Tam et al. 2013). Unfortunately, mitochondrial genomes are challenging to engineer, and mtDNA mutations are initially heteroplasmic in very low frequency, impeding both their detection and analyses of their consequences (Tsang and Lemire 2002; Gitschlag et al. 2016; Klucnika and Ma 2019).

Much of the initial work on selfish mtDNA deletions in *C. elegans* has been performed on *uaDF5*, a 3-kb deletion which encompasses four protein-coding genes (*nd1*, *atp6*, *nd2*, and *ctb-1*) and seven tRNA genes (Tsang and Lemire 2002). This deletion was formed between two 9-bp repeats and is inherited as a stable heteroplasmy in laboratory populations (Tsang and Lemire 2002; Liau et al. 2007; Gitschlag et al. 2016). The *uaDF5* deletion adversely affects fitness-related traits including productivity, longevity, and sperm motility, and yet exhibits a biased replication/transmission which increases the intracellular frequency of the deletion (Tsang and Lemire 2002; Liau et al. 2007; Gitschlag et al. 2016). Additionally, several natural isolates of the congeneric species *Caenorhabditis briggsae* harbor selfish mtDNA deletions in *nd5* (*nad5*Δ) (Clark et al. 2012). The *C. briggsae nad5Δ* deletions are detrimental for fitness and appear to persist due to a transmission bias (Estes et al. 2011; Clark et al. 2012). The severity of fitness effects and transmission/replication bias of *nad5*Δ vary with genetic background, either due to preexisting natural variation for controlling selfish mtDNA mutations or compensatory mutations (Sullins et al. 2019).

**Fig. 1:**
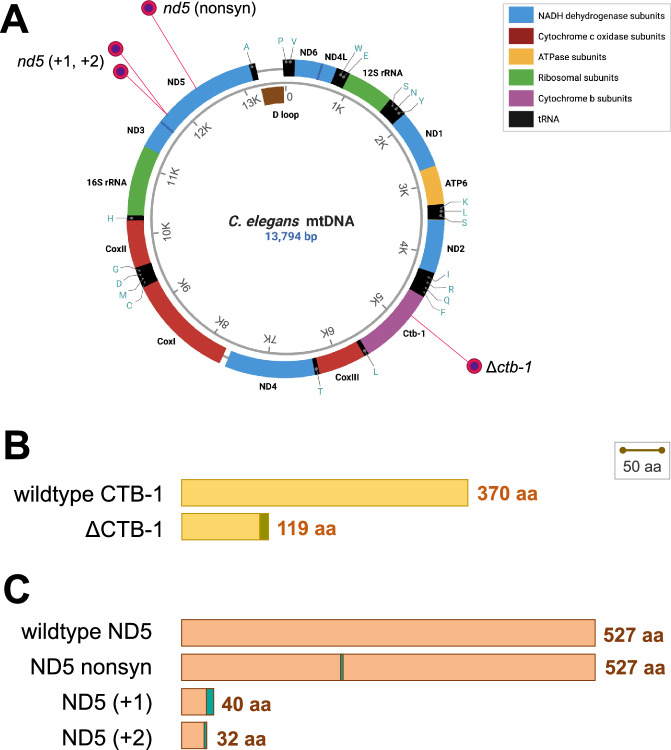
Schematic of the Δ*ctb-1* mitotype and predicted polypeptide lengths for the wildtype (wt) and mutant CTB-1 and ND5 proteins. Figures are drawn to scale. **A** A representation of the *C. elegans* mtDNA genome indicating the locations of the Δ*ctb-1* and *nd5* mutations. Image created with BioRender.com. **B** The 499 bp deletion in the *ctb-1* gene results in the contraction of the polypeptide from 370 to 119 aa. In addition, the deletion causes a frameshift in the downstream sequence resulting in 18 novel aa residues (shown in green) at the C-terminus. **C** A single T insertion in a homopolymeric run in the *nd5* gene causes a frameshift predicted to shorten the ND5 polypeptide from 527 to 40 aa, including nine novel aa residues at the C-terminus (in turquoise). A second adjacent T insertion is predicted to shorten the polypeptide further to 32 aa including a single novel aa residue at the C-terminus. Additionally depicted is the relative location of a C → T substitution which would have resulted in a Thr to Ile nonsynonymous substitution in the full-length ND5 polypeptide.

In a preceding study, we identified a spontaneously arising mitochondrial DNA deletion in an experimental evolution line (1G) (Konrad et al. 2017) from a long-term mutation accumulation (MA henceforth) experiment in *C. elegans* (Katju et al. 2015, 2018). This heteroplasmic mtDNA variant was present at 96% frequency following 346 generations of MA. In addition to this deletion which spanned 499 bp (~45%) of the *ctb-1* gene, the MA line was found to harbor four additional mtDNA mutations at the termination of the MA experiment: (i) a G → T substitution in the *tRNA*-*Asn* gene (frequency 13%), (ii) a single T insertion in a homopolymeric run within the *nd5* gene (T_8_ → T_9_; frequency 3%), (iii) an insertion of another T nucleotide in the same homopolymeric run within the *nd5* gene (T_9_ → T_10_; frequency 70%), and (iv) a C → T nonsynonymous base substitution (Thr→Ile) in the *nd5* gene (frequency 94%). These four additional heteroplasmic variants in *nd5* and *tRNA-Asn* were linked (on the same molecule) to the Δ*ctb-1* mutant allele rather than to the low-frequency *ctb-1* wildtype mtDNA (Dubie et al. 2020). The linkage of the three *nd5* variants was supported by Sanger sequencing of long PCR products (Dubie et al. 2020). PCR with one of the primers within the deleted region of *ctb-1* in heteroplasmic individuals yielded products that lacked *nd5* mutations, hence verifying that the *nd5* mutations were physically associated with the *ctb-1* deletion. Given that the Δ*ctb-1* heteroplasmic variant had the highest frequency and was easily genotyped by PCR, we referred to this set of five linked variants in MA line 1G as the Δ*ctb-1* mitotype (Fig. 1A). Dubie et al. (2020) analyzed chronologically ordered, and cryogenically archived populations of MA line 1G during the course of its laboratory evolution and delineated the sequential origin of the five mtDNA heteroplasmies comprising the Δ*ctb-1* mitotype as follows: (a) the origin of the Δ*ctb-1* variant between MA generations 25 and 51, (b) the origin of a single T insertion in the *nd5* gene between MA generations 72 and 96, (c) the origin of a C → T base substitution in the *nd5* gene between MA generations 96 and 135, (d) the origin of a second T insertion in the *nd5* gene between MA generations 221 and 300 and (e) the origin of a G → T base substitution in the *tRNA*-*Asn* gene between MA generations 221 and 300 (see Fig. 2 in Dubie et al. 2020 for the evolutionary trajectories of the secondary *nd5* mutations). We henceforth refer to the worms preserved from MA generations 71, 96, 135, 221, 300, and 350 of the 1G line as G_71_, G_96_, G_135_, G_221_, G_300_, and G_350,_ respectively, and the ancestral control as G_0_.

**Fig. 2:**
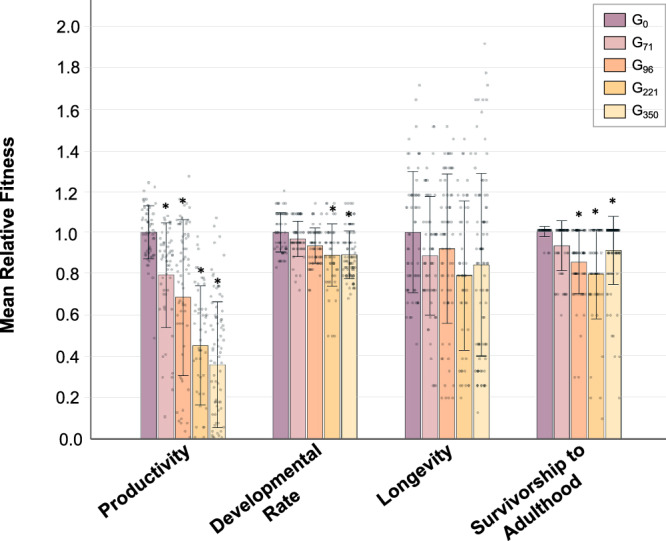
The change in four fitness traits associated with the addition of new mutations to the evolving Δ*ctb*-*1* mitotype in MA line 1G. Relative trait means ± SD of Δ*ctb-1* bearing 1G replicate lines from five generation time-points (G_0_, G_71_, G_96_, G_221_, G_350_) that were backcrossed into a wildtype N2 background. Phenotypic assays were conducted for four fitness-related traits, namely productivity, developmental rate, longevity, and survivorship to adulthood. Mean fitness values for each of the four traits were measured across four G_71_-derived lines each with 15 replicates where possible (*n* = 59-60), five G_96_-derived lines each with 15 replicates where possible (*n* = 68–74), four G_221_-derived lines each with 15 replicates where possible (*n* = 49–57), six G_350_-derived lines each with 15 replicates where possible (*n* = 87–90), and four wildtype, pre-MA ancestral control lines (G_0_) each with 15 replicates where possible (*n* = 55–60). For simplicity, the mean relative fitness value for each of the four traits in the pre-MA ancestral control (G_0_) lines was scaled to a value of 1. Asterisks indicate a significant difference (*p* ≤ 0.05) relative to the ancestral control, G_0_.

The additional missense and frameshift mutations in the Δ*ctb-1* mitotype might either be neutral, detrimental or compensatory. The compensatory effect of mutations that affect mitochondrial function was, for example, observed in *ctb-1*/*isp-1* double mutants in *C. elegans* where a mutation in *ctb-1* suppressed the slow development of a line bearing an *isp-1* mutation without improving ETC complex III function (Suthammarak et al. 2010). Given how rapidly the additional *nd5* mutations reached high intra-individual frequency in the original experiment, there is a possibility that they contribute to the transmission advantage of Δ*ctb-1*. In order to test whether the three additional *nd5* mutations contribute to the transmission advantage of the Δ*ctb-1* mitotype, we conducted an evolutionary replay experiment. Replay experiments can be used to investigate the roles of contingency and determinism in the repeatability of evolutionary outcomes (Blount et al. 2018). If the *nd5* mutations contributed to the transmission advantage of the Δ*ctb-1* mitotype even after the Δ*ctb-1* reached its maximum frequency within individuals, then the frequencies of the *nd5* mutations are expected to increase in replicate lines descended from the same starting point and under the same conditions as in the original experiment. Herein, we test how additional mutations linked to this deletion mitotype affect fitness, if they contribute to the selfish dynamics of Δ*ctb-1* or alternatively, act in a compensatory manner to ameliorate the fitness cost associated with the Δ*ctb-1* mitotype.

## Material and methods

### Archival MA strains used in this study

The focal 499 bp deletion in the mitochondrial *ctb-1* gene originated in one replicate line (1G) of a long-term *C. elegans* spontaneous MA experiment with varying population sizes (Katju et al. 2015, 2018) and was identified by whole-genome sequencing at the termination of the MA experiment at generation 409 with a heteroplasmic frequency of 96% (Konrad et al. 2017). MA line 1G was one of twenty replicate MA lines that were propagated via single hermaphrodite descent each generation (*N* = 1 individual) and had reached MA generation 346 when submitted for genome sequencing. MA line 1G was found to harbor four additional heteroplasmic mtDNA mutations relative to the ancestral control (Konrad et al. 2017): (i) a G → T base substitution in the tRNA-Asn gene (frequency 13%), (ii) a single T insertion in a homopolymeric run within the *nd5* gene (T_8_ → T_9_; frequency 3%), (iv) an insertion of a T pair in a homopolymeric run within the *nd5* gene (T_8_ → T_10_; frequency 70%), and (iv) a C → T nonsynonymous base substitution (Thr→Ile) in the *nd5* gene (frequency 94%). Given that the *ctb-1* deletion had the highest heteroplasmic frequency and was easily genotyped by PCR, we collectively referred to this set of five linked heteroplasmic mutations in line 1G as the Δ*ctb-1* mitotype for the sake of simplicity (Dubie et al. 2020). Furthermore, we used a combination of PCR and Sanger sequencing to delineate the chronological origin of these five mutations comprising the Δ*ctb*-*1* mitotype, by thawing cryogenically stored stocks of line 1G frozen at nine additional time-intervals (ancestral control G_0_ and MA generations G_25_, G_51_, G_71_, G_96_, G_135_, G_157_, G_221_, G_300_ and G_350_; Dubie et al. 2020). In this study, in order to measure the fitness consequences of each subsequent mtDNA mutation in combination with the preceding mutations, we focused on MA line 1G cryopreserved at four MA generations, namely G_71_, G_96_, G_221_, and G_350_ wherein each preceding mtDNA mutation(s) had risen to a high intracellular frequency but did not contain subsequent mtDNA mutations (Table 1). Cryopreserved stocks from each of these generations were thawed to recover nematodes for further manipulation.

**Table 1:** Frequency of each mtDNA mutation comprising the Δ*ctb-1* mitotype at different timepoints in line 1G during the course of a spontaneous mutation accumulation (MA) experiment.

MA Generation	∆*ctb-1*	*nd5 frameshift (T*_*8*_ → *T*_*9*_*)*	*nd5 C* → *T substitution*	*nd5 frameshift (T*_*9*_ → *T*_*10*_*)*
G_0_ (ancestral control)	−	−	−	−
G_71_	0.38	−	−	−
G_96_	0.90	0.27	−	−
G_135_	0.81	0.81	0.38	−
G_221_	0.98	0.78	0.88	−
G_300_	0.94	0.60	0.90	0.26
G_350_	0.87	0.14	0.91	0.78

A horizontal bar “−” represents the absence of a mutation in a particular MA generation.

### Sequestering the mtDNA heteroplasmic mutations in a wildtype background

Obligately outcrossing *C. elegans* were needed to facilitate the sequestration of mutant-bearing mtDNA in a wildtype nuclear background. To generate obligately outcrossing *C. elegans*, *fog-2* deletion-bearing lines (Δ*fog-2* henceforth) were created using InVivo Biosystems’s (formerly NemaMetrix) *C. elegans* CRISPR knockout service. Mutations resulting in a loss-of-function in *fog-2* inhibit hermaphrodite sperm production and convert the facultatively outcrossing populations into an obligately outcrossing one (Schedl and Kimble 1988). However, gene conversion events between *fog-2* and its homolog *ftr-1* have been known to restore hermaphrodite sperm production (Katju et al. 2008; Rane et al. 2010). To prevent *fog-2* gene conversion by *ftr-1*, the engineered *fog-2* deletion encompassed most of the *fog-2* open-reading frame (ORF), spanning 76 bp of intron 1 through the first 13 bp of exon 5 for a total 1106 bp of the 1212 bp ORF (Chr. V:20199390..20200491; version WS282; www.wormbase.org). After lines were received from InVivo Biosystems, they were allowed to produce progeny before the parent worms were collected for single worm DNA extraction in worm lysis buffer (90 μL 10×PCR buffer + 10 μL 10 mg/ml Proteinase K). The *fog-2* deletion was verified by PCR. First, single worm lysates were used in a PCR reaction with primers 5′—CAA AGC TTC GCT TCA TGC TC —3′ and 5′— CAG TGA TCA TGC CAA TTT ATC —3′ with expected PCR products of size 1475 bp, 369 bp and 1475/369 bp in wildtype, homozygous deletion-bearing and heterozygous lines, respectively. Lysates from lines that appeared homozygous for the deletion were then further screened by PCR using primers 5′— CAA AGC TTC GCT TCA TGC TC—3′ and 5′— TCT CCA TTG GCA TGT CAG AG—3′, with an expected 334 bp PCR product in heterozygous lines and no product in homozygous deletion-bearing lines. In homozygous deletion-bearing lines, the location of the deletion was confirmed by Sanger sequencing of the 369 bp PCR product.

Hermaphrodites recovered from cryopreserved stocks of line 1G at MA generations G_71_, G_96_, G_221_, and G_350_ were backcrossed to Δ*fog-2* males for 15 consecutive generations. After two generations of crosses to males of the mutant Δ*fog-2* strain, XX individuals were rendered homozygous for the *fog-2* null mutation and therefore incapable of self-fertilizing. This conversion of XX selfing hermaphrodites into obligate outcrossing females facilitated subsequent outcrossing with males bearing a wildtype nuclear genome. Finally, backcrossed females bearing mutant mtDNA were backcrossed with wildtype N2 males in the last two generations (generations 16 and 17), thereby replacing the functional wildtype *fog-2* allele in the females and restoring them to functional hermaphrodites with the ability to self-fertilize. After 17 generations, the proportion of the nuclear DNA from the original 1G line is estimated to be 0.5^17^ of the total nuclear DNA, or ~7.6 × 10^−6^, which virtually removes all other nuclear mutations that arose during MA. In total, four replicate lines were created from backcrossed nematodes recovered from generation G_71_ (G_71_.A – G_71_.D), five from generation G_96_ (G_96_.A – G_96_.E), four from generation G_221_ (G_221_.A – G_221_.D), and six from generation G_350_ (G_350_.C, G_350_.L, G_350_.M, G_350_.N, G_350_.T, and G_350_.U).

### Fitness assays

Phenotypic assays for four fitness-related traits (productivity, developmental rate, longevity, and survivorship to adulthood) were performed to determine the consequences of bearing each mutation combination. All fitness assays were performed using backcrossed G_71_, G_96_, G_221_, G_350_ lines and a wildtype N2 control concurrently at 20°C (Katju et al. 2015, 2018; Lee et al. 2016; Dubie et al. 2020) with 15 replicates per line.

For each replicate, a single L4 larva was isolated in a new 35 mm NGM plate seeded with OP50 prior to the line being cryopreserved using standard *C. elegans* procedures. 24h later, 10 L1 larvae offspring were transferred to a single 60 mm NGM plate seeded with *Escherichia coli* OP50 to measure survivorship. Next, a single L1 larval worm was isolated from the remaining progeny and transferred to a new 35 mm NGM plate seeded with *E. coli* OP50. This worm was used for measuring productivity, developmental rate, and longevity.

For the survivorship to adulthood assay, the 10 L1 larval worms were incubated for 48h following which the number of nematodes that had survived to adulthood were counted. The quotient of the count and 10 was then calculated to assign a survivorship score to each replicate from 0 to 1. This was then normalized by dividing the calculated survivorship score by the mean survivorship score of the N2 control prior to further analysis.

Developmental rate was measured using the single transferred L1 worm. After incubating for 30h, the nematode was visually inspected under a dissecting microscope every two hours to determine the number of hours to adulthood. A hermaphrodite was scored as having reached adulthood when its first developed egg had migrated to its uterus. The mean number of hours from L1 to adulthood of the N2 control was then divided by the observed number of hours from L1 to adulthood for the replicate to get a relative rate of development.

24h after the nematode used in the previous assay had reached adulthood, and every 24h thereafter for eight days, the adult nematode was transferred to a new plate. The plate from which the nematode had been transferred was then incubated for an additional 24h before being stored at 4°C for 30 days. After 30 days, the plates were stained with 200 µL of 0.1% toluidine blue solution and the number of offspring counted. The total count between all eight plates was recorded as the raw productivity which was then normalized by dividing by the mean raw productivity of the N2 control.

Longevity was measured by further transferring the nematode used above to a new plate as a nine-day adult worm. The worm was then inspected every day to determine if it was still alive. A nematode lacking movement and pharyngeal pumping was then gently prodded with a worm pick. The day a nematode did not respond to prodding was recorded as its day of death. Raw longevity was calculated as the number of days between plating the L1 for the time to adulthood assay and the day of death. A relative longevity score was then calculated by dividing the raw longevity by the mean raw longevity of the N2 control.

### Mutation accumulation (MA) “replay” experiments

In order to test if the four high-frequency mtDNA heteroplasmic mutations in line 1G during the MA experiment exhibit selfish drive, mutation-bearing nematodes were propagated in populations with reduced inter-individual selection (mirroring the original MA experiment) while tracking the intra-individual dynamics of each mtDNA variant over time. Cryopreserved nematodes from the original MA experiment were recovered from generations in which the heteroplasmic frequency of a mutation of interest was low to intermediate (Table 1). A single third-generation descendant from thawed worms of cryopreserved stocks of four MA generations (G_71_, G_96_, G_135_, and G_300_) was used to initiate the replay experiments without contributions of maternal and grandmaternal environmental effects (Lynch 1985). This single L4 (fourth larval stage) hermaphrodite was isolated onto a 35 mm NGM plate seeded with a bacterial lawn of *E. coli* OP50 and marked as the parental generation (P) for the “replay” experiment. 25 randomly selected F_1_ progeny of the P worm were individually isolated as L4 larvae onto NGM/OP50 plates to establish 25 replicate lines. This protocol yielded 100 experimental lines comprising the replay experiments (25 replicates × four mutations). The P hermaphrodite was collected, and DNA was extracted by single worm lysis using the Sigma Extract-N Amp kit. Following establishment, the 100 replay lines were experimentally evolved by propagating them via single-worm bottlenecks (*N* = 1 individual) every four days for 10 successive generations. Additionally, the F_5_ and F_10_ progenitor worms were also collected as day 2 adults and their DNA extracted by single worm lysis. All experimental lines were incubated at 20°C for the entire length of the replay experiments. The change in the frequency of mtDNA mutations during the replay experiments was tested using a *t-*test between generations zero and 10 and a paired *t*-test between generations five and 10, using a Holm-Bonferroni adjustment of *p*-values for multiple tests (Aickin and Gensler 1996).

### Calculation of the frequency of heteroplasmic mutations

The heteroplasmic frequency of the ∆*ctb-1* deletion was determined by digital droplet PCR (ddPCR) using previously established methods (Dubie et al. 2020) in the parental worms from generations 0, 5, and 10 of the replay experiment. Briefly, each multiplexed reaction was performed with 2.2 µL of single worm lysate diluted 1:100 with molecular grade water, 11 µL Bio-Rad ddPCR supermix for probes (no dUTP) (catalog # 1863024), 1.1 µL Bio-Rad custom ddPCR copy-number assay with a 6-carboxyfluorescein (FAM) fluorophore targeting a region within the *ctb-1* deletion (assay ID: dCNS821148280), 1.1 µL Bio-Rad custom ddPCR copy-number assay with a 5′-hexachlorofluorescein phosphoramidite (HEX) fluorophore targeting a conserved region of *cox-1* (assay ID: dCNS484374227), and 6.6 µL molecular grade water. The FAM assay was used to calculate the concentration of wildtype, intact *ctb-1* mtDNA templates in each sample. The HEX assay was used to calculate the concentration of mtDNA templates per sample. For each reaction droplet, separation thresholds and concentration of each template were calculated automatically by Quantisoft (Bio-Rad Laboratories) and the heteroplasmic frequency of the deletion was calculated as:

$$f(\Delta {\rm{mtDNA}})=\frac{{\rm{total\; mtDNA\; concentration}}-{\rm{wildtype\; mtDNA\; concentration}}}{{\rm{total\; mtDNA\; concentration}}}$$

The heteroplasmic frequencies of all *nd5* mutations were determined by examining relative fluorescence in chromatograms generated by Sanger sequencing as previously described in Dubie et al. (2020). Briefly, three independent PCR products were generated from each single worm lysate using standard PCR protocols and primers designed to amplify a region of *C. elegans nd5* which contained all three mutations (forward primer: 5′− TCA TCT TCA TCT TGG GAG GAT TT − 3′; reverse primer: 5′− GTG TCC TCA AGG CTA CCA CC −3′). PCR samples were then sequenced by Eton Biosciences using the forward primer for the *nd5* frameshift mutations and the reverse primer for the *nd5* nonsynonymous base substitution. The generated .ab1 chromatogram files were processed in Chromas (http://technelysium.com.au/wp/chromas/) to generate images that were subsequently processed in FIJI to calculate the heteroplasmic frequency of each mutation.

To measure the heteroplasmic frequency of the T_8_ → T_9_ frameshift mutation in *nd5*, the peak heights at the first and second double peak after the poly-T run at mtDNA:11,778 were measured with FIJI (Schindelin et al. 2012) and substituted into the following formula:

$$f({T}_{8}\to {T}_{9})=\frac{(({T}_{9}/({A}_{1}+{T}_{9}))+({A}_{2}/({A}_{2}+{G}_{1})))}{2}$$

where *A*_*1*_ and *T*_*9*_ are the heights of overlapping peaks at the ninth position of the poly-T run and *A*_*2*_ and *G*_*1*_ are the heights of overlapping peaks just after the poly-T run.

When the T_9_ → T_10_ insertion is present in addition to the T_8_ → T_9_ insertion, the frequencies of both T_8_ → T_9_ and T_9_ → T_10_ mutations were calculated as follows:

$$f\left({T}_{8}\to {T}_{9}\right)=\frac{A}{A+G+{T}_{10}}$$

and

$$f\left({T}_{9}\to {T}_{10}\right)=\frac{{T}_{10}}{A+G+{T}_{10}}$$

where *A*, *G* and T_10_ are the corresponding bases in the first triple peak downstream of the insertion.

Lastly, the heteroplasmic frequency of the nonsynonymous substitution in *nd5* was determined using measurements of the double peak at mtDNA:12,304 with the following formula:

$$f({nonsynonymous\; substitution})=\frac{T}{T+C}$$

In each case, the calculated frequencies from the three sequenced PCR reactions were then averaged to yield a final estimate of the heteroplasmic mutation frequency of each sample.

### Calculation of mitochondrial copy-number

Average mtDNA copy-number per cell was measured using ddPCR. In all cases, nematodes were recovered from cryopreserved stocks and grown on 60 mm NGM plates seeded with *E. coli* OP50. DNA from a single worm was extracted using the Sigma Extract-N Amp kit (catalog # XNAT2-1KT). The single worm lysate was diluted 1:50 in molecular grade water and added to a multiplexed reaction containing 2.2 µL diluted single worm lysate, 11 µL Bio-Rad ddPCR supermix for probes (no dUTP) (catalog # 1863024), 1.1 µL Bio-Rad custom ddPCR copy-number assay with a FAM fluorophore targeting the nuclear gene *actin-2* (assay ID: dCNS440514568), 1.1 µL Bio-Rad custom ddPCR copy-number assay with a HEX fluorophore targeting *cox-1* (assay ID: dCNS484374227), and 6.6 µL molecular grade water. The FAM and the HEX assay were used to calculate the concentration of the nDNA and mtDNA templates per sample, respectively. For each reaction, droplet separation thresholds and concentration of each template were calculated automatically by Quantisoft (Bio-Rad Laboratories) and relative mtDNA copy-number was calculated as:

$$\frac{2({\rm{mtDNA\; concentration}})}{{\rm{nDNA\; concentration}}}$$

We measured average mtDNA copy-number in worms from the N2 ancestral control, and MA generations G_71_, G_96_, G_135_, G_221_, G_300_, and G_350_ lines as well as the backcrossed G_71_, G_96_, G_221_, and G_350_ lines.

Two of the MA lines appeared to have unusually elevated (G_135_) or reduced (G_221_) mtDNA copy-number (see Results). To ensure that these were indeed changes in mtDNA copy-number and not variation in the copy-number of *actin-2*, an additional duplexed reaction was performed for three samples per MA line, replacing the Bio-Rad custom ddPCR copy-number assay with a FAM fluorophore targeting *daf-3* on the X chromosome (assay ID: dCNS736258201) and a Bio-Rad custom ddPCR copy-number assay with a HEX fluorophore targeting the same region as the previous *actin-2* probe (assay ID: dCNS257340490). The results confirmed that there was no copy-number variation in the region targeted by the *actin-2* probe. However, this region is duplicated whereas the region targeted by the *daf-3* probe is single-copy. We therefore report the mtDNA copy-number normalized to *daf-3* in the results.

## Results

The effect of the four mutations impinging on polypeptide length and sequence are schematically displayed in Fig. 1 and Fig. S1. The first mutation, the Δ*ctb-1* deletion, resulted in the abbreviation of the wildtype protein by ~68%, from 370 to 119 aa residues (Fig. 1B). In addition, this deletion resulted in a frameshift which introduced 18 novel aa residues at the terminal end of the contracted polypeptide (Fig. S1A). The second mutation, a single T insertion in *nd5* engendered a similarly drastic effect on both the length and amino acid sequence of the wildtype polypeptide. It resulted in the abbreviation of the ND5 wildtype polypeptide by ~92%, from 527 to 40 aa residues in conjunction with the introduction of nine novel residues at the end of the new terminal region (Fig. 1C; Fig. S1B). The third mutation, a base substitution which would have resulted in a nonsynonymous mutation (Thr→Ile) in the full length *nd5*, is downstream of the predicted coding sequence in the frameshifted *nd5* gene (Fig. 1C; Fig. S1B). The fourth mutation, a second T insertion in the same homopolymeric run as the first T, further reduced the resultant ND5 protein to 32 aa residues (a contraction of the *nd5* protein by ~94%) and introduced one novel residue at the terminal end of the mutant protein (Fig. 1C; Fig. S1B). The frequencies of the mtDNA mutations in MA line 1G at different timepoints and in the backcrossed lines employed in the fitness assays are listed in Table 1 and Table S1, respectively.

### Significant decline in fitness with the addition of subsequent mitochondrial mutations

In order to quantify the fitness effects of each incremental addition of a new mtDNA variant to this evolving mitotype, we assessed the fitness of the Δ*ctb-1* mitotype from MA generations G_71_, G_96_, G_221_, and G_350_ in a wildtype N2 genetic background (see backcrossing design in the **Material and Methods** section). The productivity, developmental rate, longevity, and survivorship of backcrossed mtDNA mutation-bearing lines were compared to each other and the cryopreserved ancestral control G_0_. For each fitness trait, a nested ANOVA analysis found a significant variance component for productivity (*F*_*s*_’ = 55.12; *p* < 0.0001), developmental rate (*F*_*s*_’ = 19.42; *p* < 0.0001), longevity (*F*_*s*_’ = 2.65; *p* < 0.05), and survivorship (*F*_*s*_’ = 17.04; *p* < 0.0001) among the five generation times (G_0_, G_71_, G_96_, G_221_, and G_350_) (Table S2). There was no significant among-replicates divergence within groups (five generation times listed above) for productivity, developmental rate, and longevity. However, a significant among-replicates divergence was noted for survivorship (*F*_*s*_ = 3.65; *p* < 0.0001) (Table S2). The among-replicate variation in survivorship could be due to differences in the genetic background, the frequency of mtDNA mutations or mtDNA copy-number.

The backcrossed lines showed a monotonic decline in mean productivity (Fig. 2) with G_71_, G_96_, G_221_, and G_350_ derived lines producing an average 247, 214, 141, and 112 progeny, respectively, compared to the mean productivity of 312 progeny for the G_0_ ancestor. These numbers correspond to relative mean productivity of ~0.79, 0.69, 0.45 and 0.36 for G_71_, G_96_, G_221_, and G350, respectively (Fig. 2), all of which are significantly reduced compared to the relative mean productivity of 1 for the G_0_ ancestor (Table S3). The initial decline in productivity coincides with an increase in the frequency of the *ctb-1* deletion, which had reached a 90% frequency by G_96_ (Table 1). Between generations G_71_ and G_96,_ the first *nd5* frameshift mutation becomes detectable at an estimated frequency of 27% (Table 1). However, the additional 11% decline in relative mean productivity from G_71_ to G_96_ was not significant (Table S3). There was another significant decline (23%) in productivity from G_96_ to G_221_ that coincided with two *nd5* mutations reaching high frequencies, the first frameshift mutation (78%) and a downstream substitution (88%) (Table S3). The 9% reduction in relative mean productivity from G_221_ to G_350_, the period during which a secondary *nd5* frameshift mutation replaced the preceding one, was not significant (Table S3).

The backcrossed lines took 0.8%, 4.3%, 8.9%, and 11.7% longer to reach reproductive maturity than the G_0_ ancestor at generations G_71_, G_96_, G_221_, and G_350_, respectively. This trend represents a more gradual rate of decline for development relative to that for productivity (Fig. 2). Both the gradual rate and absolute change in mean developmental rate (~12%) across 350 generations demonstrates that this trait is more robust to an increase in mutation load relative to productivity. However, there was an observable significant delay in development by generation G_221_ onwards which corresponds to high frequencies of the *ctb-1* deletion, the first *nd5* frameshift mutation, and the *nd5* substitution (Table S3). Although the ANOVA analyses suggested that mitotype had a significant effect on longevity (Table 2), no pairwise comparison between generations showed a significant difference in longevity (Table S3). The average survivorship of all backcrossed lines from G_96_ onwards was significantly lower than the ancestral control, G_0_ (Table S3; Fig. 2). Relative mean survivorship also appears to increase from 79.9% at generation G_221_ to 91.1% in generation G_350_.

**Table 2:** Test of pair-wise differences in average mtDNA copy-number in line 1G cryopreserved at different generations of the MA experiment (ancestral control G_0_, G_71_, G_96_, G_135_, G_221_, G_300_, G_350_) using the Tukey–Kramer HSD method of multiple comparisons among pairs of means.

*Ancestral lines*							
	G_0_	G_71_	G_96_	G_135_	G_221_	G_300_	G_350_
G_0_	–	0.443	0.419	0.500	0.433	0.457	0.433
G_71_	0.333	–	0.349	0.443	0.366	0.394	0.366
G_96_	0.412	0.079	–	0.419	0.336	0.365	0.336
G_135_	1.336*	1.003*	0.924*	–	0.433	0.457	0.433
G_221_	0.809*	1.141*	1.220*	2.145*	–	0.382	0.354
G_300_	0.265	0.068	0.147	1.071*	1.074*	–	0.382
G_350_	0.515*	0.183	0.104	0.820*	1.324*	0.251	–

The differences in mtDNA copy-number for all subsequent generation times are scaled to mtDNA copy-number at generation G_0_ (ancestral control).

Absolute differences among all pairs of trait means *i* and *j* are listed below the diagonal and their critical MSD*ij* values above the diagonal. A pair of means is declared significantly different at *α* = 0.05 if their absolute difference equals or exceeds their MSD values (indicated by an asterisk).

^∗^ A pair of means that are significantly different at an experiment-wise error rate of *α* = 0.05.

The creation of backcrossed lines containing the heteroplasmic mtDNA mutations results in sequential bottlenecks which minimizes the efficiency of individual selection and can reveal important intra-individual dynamics. However, it also means that the heteroplasmic frequencies in the backcrossed lines do not exactly mirror the heteroplasmic frequencies in the original MA lines (Table S1). For example, the *ctb-1* deletion which was at a frequency of 0.38 in generation G_71_ increased to an average of 0.72 during the backcrossing regime (Table S1). The T_8_ → T_9_ frameshift mutation in *nd5*, which was at a frequency of 0.27 in generation G_96_ declined to 0.14 in the backcrossed lines. Although the shifting frequencies of the mtDNA heteroplasmies pose a challenge in drawing strong conclusions about their fitness consequences, the results contradict the hypothesis that mutations that arose subsequent to the *ctb-1* deletion are compensatory.

### Transmission advantage of additional heteroplasmic mutations comprising the Δ*ctb-1* mitotype

In the original MA experiment, the mitochondrial mutations in line 1G rapidly reached frequencies nearing fixation (Dubie et al. 2020). In order to test the reproducibility of the frequency trajectories of the four mtDNA mutations in this MA line, we established 4 × 25 replicate lines derived from single hermaprodites isolated at different time-points of the original MA experiment, namely generations G_71_, G_96_, G_135_, and G_300_. These 4 × 25 replicate lines, descended from individuals in the original MA experiment without backcrossing into a wildtype nuclear genetic background, were propagated at *N* = 1 hermaphrodite per generation for ten additional generations. This extreme bottlenecking regime is expected to negate inter-individual competition, and thereby reveal intra-individual population dynamics of the different mutations comprising the Δ*ctb-1* mitotype. The dynamics of the heteroplasmic mutations in these “replay” experiments are expected to be influenced by both the individual mutation and the nuclear genetic backgound. Hence, these experiments were designed to test if the mtDNA mutations in the *Δctb-1* mitotype reached high intracellular frequency by random genetic drift or a transmission advantage.

**Fig. 3:**
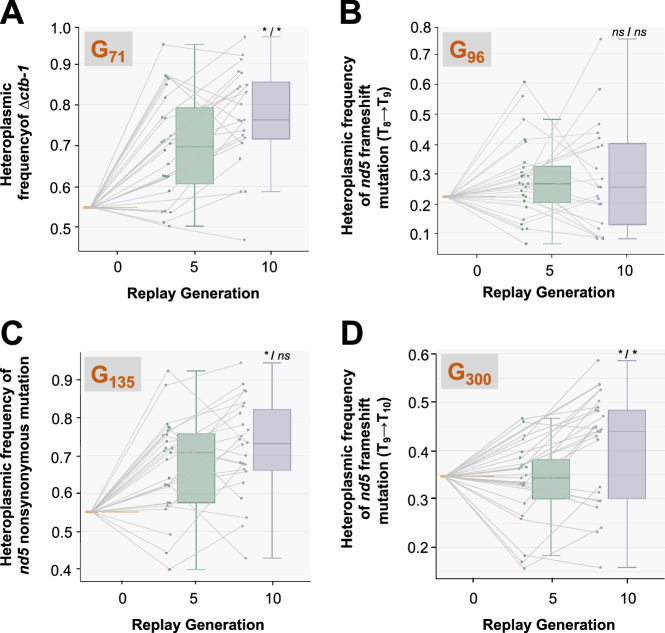
The change in frequencies of heteroplasmic mutations during a 10-generation replay experiment. **A** Change in the frequency of the *ctb-1* deletion from generation G_71_. **B** Change in the frequency of the T_8_ → T_9_ frameshift insertion in *nd5* from generation G_96_. **C** Change in the frequency of the C → T base substitution in *nd5* from generation G_135_. **D** Change in the frequency of the T_9_ → T_10_ frameshift insertion in *nd5* from generation G_300_. For each mutation, 25 replicates lines were propagated as bottlenecked populations, all originating from the same generation G_0_ parent whose heteroplasmic frequency is reported as a single bar at generation 0 of the replay experiment. For Generation 10 of the replay experiment, the symbol before the “/” indicates a comparison to Generation 0 of the replay experiment, whereas the symbol after the “/” represents the paired comparison to Generation 5 of the replay experiment (asterisk indicates a significant difference with *p* ≤ 0.05; *ns* represents no significant difference). Solid lines within the boxplots represent the median heteroplasmic frequency. The lines connecting the dots represent the heteroplasmic frequency trajectories of individual replicate lines at different timepoints of the replay experiment.

In Dubie et al. (2020), the Δ*ctb-1* deletion was observed at a frequency of 0.38 at generation G_71_ (Table 1). However, its frequency was determined to be 0.55 in the ancestor of the 25 replicate G_71_ lines (Fig. 3A). The average frequency of the deletion rose to 0.77 (median 0.76) after 10 generations (Fig. 3A). The heteroplasmic frequency of the Δ*ctb-1* deletion was significantly higher at generation 10 (*t* = 8.33, *p* = 6.26 × 10^−8^; Holm-Bonferroni adjusted *p* = 5.01 × 10^−7^) relative to generation 0. Moreover, the increase in the heteroplasmic frequency of the deletion was also significant between generations five and 10 (paired *t*-test: *t* = 3.40, *p* = 0.003; Holm-Bonferroni adjusted *p* = 0.017; Fig. 3A). The directional change in the frequency of the Δ*ctb-1* deletion during the replay experiment suggests a replication/transmission advantage.

The Δ*ctb-1* deletion reached a frequency of >0.90 in one of the replicate lines after five generations, and in two lines after 10 generations. The Δ*ctb-1* deletion was typically observed to increase in frequency between generations five and 10 in lines when its frequency was <0.80 (*n* = 15) at generation five, but not if its frequency was >0.80 at generation five (*n* = 6). At generation G_96_ of the original MA experiment, the Δ*ctb-1* deletion had reached a frequency of 0.90 and accrued an additional mutation at a frequency of 0.27, namely a frameshift in *nd5* caused by the insertion of a T nucleotide in a run of eight Ts (T_8_ → T_9_) (Table 1). The G_96_ ancestor of the 25 replicates had the frameshift mutation at a frequency of 0.22. This heteroplasmic T_8_ → T_9_ mutation was present in an average frequency of 0.28 (median 0.26) after 10 generations. There was no significant difference in the heteroplasmic frequency of the T_9_ frameshift mutation between generations zero and 10 (*t* = 1.54, *p* = 0.13; Holm-Bonferroni adjusted *p* = 0.26) and a paired *t* test between generations five and 10 was also nonsignificant (*t* = 1.24, *p* = 0.91; Fig. 3B). The frequency of the T_9_ frameshift mutation displayed greater variation among the replicate lines at generation 10_,_ with one replicate line exhibiting a T_9_ frequency as high as 0.75 in comparison to <0.10 frequency in other replicate lines. This frequency variation of the T_9_ variant in *nd5* across 10 generations suggests that it is dominated by genetic drift.

At generation G_135_ of the MA experiment, the frequency of the Δ*ctb-1* deletion and the first *nd5* frameshift were both estimated at 0.81 (Dubie et al. 2020; Table 1). Additionally, the MA line at generation G_135_ has further acquired a novel C → T substitution in the *nd5* gene downstream of the initial frameshift mutation, at a frequency of 0.38 (Dubie et al. 2020). The *nd5* substitution was present at 0.55 frequency in the generation G_135_ ancestor of the 25 replicate lines and further increased to an average frequency of 0.72 after 10 generations (Fig. 3C). The difference in the frequency of *nd5* substitution was significant between generations zero and 10 (*t* = 6.61, *p* = 9.40 × 10^−7^; Holm-Bonferroni adjusted *p* = 6.58 × 10^−6^). However, a paired *t*-test between generations five and 10 was nonsignificant (*t* = 1.67, *p* = 0.11; Holm-Bonferroni adjusted *p* = 0.33). The frequency increase of the C → T heteroplasmy between generations zero and 10 suggests that this base substitution contributes to the transmission advantage of the Δ*ctb-1* mitotype. There was no average change in the *nd5* base substitution frequency between generations five and 10 in replicate lines where the substitution was in >0.70 (*n* = 13) frequency at generation five. However, in replicate lines with a substitution frequency <0.70 (*n* = 12), the frequency significantly increased by 0.09 (paired *t*-test, *t* = 2.33, *p* = 0.042).

At generation G_300_ of the MA experiment, the frequency of the Δ*ctb-1* deletion and *nd5* C → T substitution was 0.94 and 0.90, respectively (Dubie et al. 2020; Table 1). The frequency of the *nd5* T_9_ variant was 0.60, having declined from a frequency of 0.81 at generation G_135_. In addition to the T_9_ variant, a second insertion of a T nucleotide (T_9_ → T_10_) in the same homopolymeric run was observed at a frequency of 0.26. The combined frequency of the two frameshift mutations in the same homopolymeric run within *nd5* was 0.86. The G_300_ ancestor of the 25 replicate lines possessed the T_10_ and T_9_ frameshift at a frequency of 0.35 and 0.59, respectively (combined frequency of T_9_ + T_10_ at 0.94 frequency). Following the first five generations, the 25 replicate lines had T_10_ and T_9_ frameshift variants at an average frequency of 0.34 and 0.59, respectively (Fig. 3D). However, following 10 generations, the frequency of the T_10_ frameshift had increased to 0.40 with a concomitant decline in the frequency of the T_9_ variant to 0.51 (Fig. 3D). Although the frequency of the T_10_ frameshift was not significantly higher than the starting frequency of 0.35 after 10 generations (*t* = 2.45, *p* = 0.023; Holm-Bonferroni adjusted *p* = 0.09), a paired *t*-test between generations five and 10 was significant (*t* = 4.13, *p* = 0.0005; Holm-Bonferroni adjusted *p* = 0.003). The average combined frequency of the T_9_ and T_10_ frameshifts in *nd5* remained stable during the 10 generations of experimental evolution, and as in the original MA experiment, the T_9_ variant was gradually replaced by T_10_. The frequency of the *nd5* base substitution also remained stable in the 25 replicate lines over the 10 generations, with an average frequency of 0.92 and 0.93 after five and 10 generations, respectively. The frequency increase of the T_9_ → T_10_ heteroplasmy suggests that the second frameshift in *nd5* contributes to the transmission advantage of the Δ*ctb-1* mitotype.

### mtDNA copy-number variation in ancestral and backcrossed lines

**Fig. 4:**
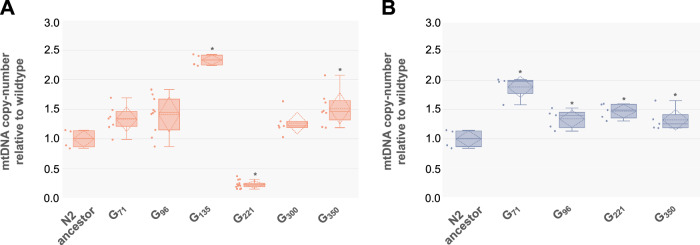
Variation in mtDNA copy-number in the original MA lines and backcrossed lines bearing the *Δctb-1* deletion and associated *nd5* mutations. Solid and dashed horizontal lines within the boxplot denote the median and mean value, respectively. **A** A boxplot of relative mtDNA copy-number in line 1G cryopreserved at different generations of the MA experiment (ancestral N2 control, G_71_, G_96_, G_135_, G_221_, G_300_, G_350_). **B** A boxplot of relative mtDNA copy-number in heteroplasmic lines carrying mitotypes from different generations (ancestral N2 control, G_71_, G_96_, G_221_, G_350_) of MA line 1G backcrossed into a wildtype N2 background. Asterisks indicate a significant difference (*p* ≤ 0.05) relative to the wildtype N2 ancestral control.

Deleterious mtDNA mutations are frequently associated with increased mtDNA copy-number. We measured relative mtDNA copy-number at multiple time-points in both the original MA lines and in backcrossed lines with a wildtype (N2) nuclear genetic background. The original MA lines, in which both nuclear and mitochondrial mutations are present, vary considerably in relative mtDNA copy-number. Three of the five generations of the 1G MA line (G_135_, G_221_, and G_350_) exhibit significantly greater mtDNA copy-number than their ancestral control (Fig. 4A, Table 2). Surprisingly, generation G_221_ had extremely reduced mtDNA copy-number comprising a minor fraction (19%) of that in the ancestral control (Fig. 4A). A repeat experiment with generation G_221_ worms confirmed the results of this relatively low mtDNA copy-number. The determination of mtDNA copy-number is based on a comparison of mtDNA and nuclear DNA probes. We initially used an *actin-2* nuclear DNA probe. However, the *actin-2* locus is a recent gene duplicate and if there were additional copy-number changes in the nuclear locus, it would influence the relative mtDNA copy-number estimates. For example, additional duplications of the *actin-2* target sequence would have the effect of diminishing the estimated mtDNA copy-number. The use of an additional single-copy nuclear probe, *daf-3*, showed that there was no significant difference in *actin-2* copy-number between G_0_ ancestor and generation G_221_. With the exception of generation G_221_, mtDNA copy-number was, on average, 1.4-fold greater than the ancestral control in the other MA generations (G_71_, G_221_, G_300_, G_350_)_._ In worms where the mtDNA from different MA generations was backcrossed into the wildtype (N2) nuclear DNA background, mtDNA copy-number was, on average, 1.5-fold greater than the ancestral control G_0_ (Fig. 4B, Table 3).

**Table 3:** Test of pair-wise differences in average mtDNA copy-number of mitotypes from different generations (ancestral control G_0_, G_71_, G_96_, G_221_, G_350_) of MA line 1G backcrossed into N2 using the Tukey–Kramer HSD method of multiple comparisons among pairs of means.

*Backcrossed lines*					
	G_0_	G_71_	G_96_	G_221_	G_350_
G_0_	–	0.333	0.310	0.310	0.294
G_71_	0.892*	–	0.365	0.365	0.351
G_96_	0.341*	0.551*	–	0.344	0.329
G_221_	0.474*	0.418*	0.133	–	0.329
G_350_	0.400*	0.491*	0.059	0.073	–

The differences in mtDNA copy-number for all subsequent generation times are scaled to mtDNA copy-number at generation G_0_ (ancestral control).

Absolute differences among all pairs of trait means *i* and *j* are listed below the diagonal and their critical MSD*ij* values above the diagonal. A pair of means is declared significantly different at *α* = 0.05 if their absolute difference equals or exceeds their MSD values (indicated by an asterisk).

^∗^A pair of means that are significantly different at an experiment-wise error rate of *α* = 0.05.

## Discussion

Deletions in *C. elegans* mtDNA usually entail both a significant fitness cost and a replication advantage allowing them to persist in laboratory populations (Tsang and Lemire 2002; Gitschlag et al. 2016; Dubie et al. 2020; Meshnik et al. 2022; Sequeira et al. 2024). One of these selfish mtDNA deletions in *ctb-1* rose rapidly to high frequency during an MA experiment and remained in >80% of the total mtDNA for >250 generations of single individual bottlenecking (Dubie et al. 2020). During the period of experimental evolution, the Δ*ctb-1* mitotype acquired additional mutations, especially in *nd5*, which also rose to high frequencies and were found to be linked to the original *ctb-1* deletion at the termination of the MA experiment. Two of the *nd5* mutations were single nucleotide insertions that occurred sequentially in the same homopolymeric run, first increasing the length of the run from T_8_ → T_9,_ and subsequently, from T_9_ → T_10_. This homopolymeric run in *nd5* is a mutational hotspot that accounted for 16% of the mtDNA mutations during MA (Konrad et al. 2017). The T_8_ → T_9_ and T_9_ → T_10_ insertions result in premature stop codons that shorten the total length of the polypeptide from 527 aa to 40 and 32 aa, respectively. Additionally, a C → T substitution occurred 526 bp downstream of the T_8_ site. The combined frequency of the frameshifts (T_9_ + T_10_), and independently that of the downstream substitution became equal to the frequency of the *ctb-1* deletion. A combination of long PCR and DNA sequencing suggested that in the terminal generation of the original MA experiment (Katju et al. 2015, 2018), every mtDNA molecule that carried a *ctb-1* deletion also carried either the T_9_ or T_10_ frameshift and the downstream base substitution (Dubie et al. 2020). This raised the question of why the Δ*ctb-1* mitotype ended up with additional and possibly deleterious mutations in *nd5*. There are at least three possibilities: (i) a chance association between the deletion and additional mutations combined with intracellular random genetic drift, (ii) the secondary mutations ameliorated the fitness cost of the original *ctb-1* deletion, and (iii) the additional mutations also possess selfish properties. We did not investigate the effect of a mutation in tRNA-Asn, which arose later in the original MA experiment and was never found in a heteroplasmic frequency greater than 13% (Dubie et al. 2020). We used a line of *C. elegans* that had been cryopreserved at different timepoints throughout 350 generations of MA to dissect the impact that this series of mitochondrial mutations had on a previously characterized selfish mitotype. Using nematodes recovered from this experiment, we were able to demonstrate that none of the additional mtDNA mutations compensated for the deleterious effects of the preceding mutations. Furthermore, the evidence suggests that some of the *nd5* mutations can contribute to the selfish genetic drive of the Δ*ctb-1* mitotype in the intracellular environment in which they arose.

Mutations that cause severe mitochondrial dysfunction can be partially compensated for by additional mtDNA mutations. For instance, the large reduction in fecundity associated with an *isp-1* mutation can be partially suppressed by a secondary mutation in *ctb-1* (Feng et al. 2001). We tested the hypothesis that following the original *ctb-1* deletion, subsequent mtDNA mutations improved fitness. In *C. elegans*, productivity appears to be the quantitative fitness trait that is most sensitive to MA and is expected to be most strongly associated with fitness (Katju et al. 2015, 2018). We observed a monotonic decline in productivity and developmental rate associated with the accumulation of each subsequent mtDNA mutation that arose after the original *ctb-1* deletion which suggests that the secondary *nd5* mutations do not ameliorate the fitness cost associated with the deletion. However, the fitness traits were measured in a common genetic background (N2) to dissociate the fitness effect of the mtDNA mutations from nuclear mutations that accumulated during MA. This also limits the interpretation of the results to the common genetic background, and it remains a possibility that the fitness contributions of the secondary mutations in *nd5* were more beneficial or less detrimental in the genetic background where they originated.

Experimental evolution studies where individuals and populations can be cryogenically preserved at different time-points during the experiment offer an inherent advantage to investigations into the repeatability and convergence of evolutionary trajectories. In the original MA experiment, the *ctb-1* deletion and subsequent *nd5* mutations rapidly rose to, and remained in high frequency with the exception of the T_8_ → T_9_ insertion which was replaced by the new T_9_ → T_10_ frameshift mutation. Nonetheless, after the appearance of the T_9_ → T_10_ insertion_,_ the combined frequency of T_8_ → T_9_ and T_9_ → T_10_ insertions remained high and indistinguishable from the frequency of the *ctb-1* deletion. Furthermore, the evidence from long PCR suggests that the additional mutations are physically linked to the *ctb-1* deletion (Dubie et al. 2020). Would the mtDNA mutations that arose during the MA experiment rise in frequency again if we restarted the experimental evolution process from intermediate frequencies of each of the mtDNA mutations? In the event that the mtDNA mutations did not have a transmission or replication advantage, we expect that the average intra-individual frequency of the mutation would not increase, and perhaps even decrease if strongly detrimental to fitness, during single individual bottlenecks across multiple generations. This may be the case for the first *nd5* frameshift mutation (T_8_ → T_9_). In contrast, the *ctb-1* deletion, and both the C → T substitution and the T_9_ → T_10_ frameshift displayed an average increase in frequency during replay experiments involving 10 generations of single individual bottlenecks. The consequences of both the *nd5* frameshifts and base substitution on the translated products are not known. Assuming the conventional start codon for *nd5*, the peptide sequence is shortened from 527 to 40 aa and 32 aa following the first and second frameshift mutation, respectively. In these instances, the downstream base substitution should be inconsequential for the translation of the shortened *nd5* peptide. Alternatively, the frameshift mutation might split the *nd5* coding sequence into two ORFs with translation restarting downstream of the frameshift mutations, in which case the majority of the *nd5* protein is translated in-frame and the C → T (Thr → Ala) base substitution is nonsynonymous.

The increase in the frequency of the *ctb-1* deletion through repeated bottlenecks has been demonstrated before (Dubie et al. 2020). Replication advantage may be a general feature of mtDNA deletions in *C. elegans* and *C. briggsae* as several of these increase in frequency under relaxed selection or remain in high frequency for extensive periods despite a demonstrable fitness cost (Tsang and Lemire 2002; Howe and Denver 2008; Clark et al. 2012; Meshnik et al. 2022; Sequeira et al. 2024). The simplest hypothesis for the replication advantage of mtDNA deletions is that shorter mtDNA molecules replicate faster (Wallace 1992; Diaz et al. 2002). It can be argued that the rate-limiting step for replication of mtDNA genomes is the initiation of replication, and not the time it takes to complete one round of replication. However, because the *C. elegans* mtDNA is replicated by a rolling circle mechanism (Lewis et al. 2015), the completion of replication of a single mitochondrial genome is intrinsically coupled with the initiation of another round of replication which could give smaller genomes a replicative advantage. Still, direct evidence for replicative advantage of shorter genomes in *C. elegans* is lacking (Gitschlag et al. 2016).

Cells with a high proportion of a detrimental mtDNA mutation over-replicate their mtDNA to compensate for impaired mitochondrial function and consequently have greater mtDNA copy-number than cells with wildtype mtDNA (Capps et al. 2003; Gitschlag et al. 2016). Importantly, random mtDNA replication maintaining sufficient copy-number of wildtype mtDNA can result in the proliferation of mutant mtDNA (Chinnery and Samuels 1999). Thus, selfish mtDNA mutations can be viewed as exploiting mtDNA copy-number control to increase in frequency within cells (Gitschlag et al. 2016). mtDNA copy-number was increased in worms harboring the *ctb-1* deletion, both in the original MA lines and in lines with the Δ*ctb-1* mitotype in an N2 nuclear genetic background. There are two key observations from the mtDNA copy-number analyses in addition to the general increase in mtDNA quantity. First, there is much greater variation in total mtDNA copy-number between MA lines from different generations than in the backcrossed lines, presumably due to differences in the nuclear genetic background. This variation includes a precipitous drop in relative mtDNA quantity in generation G_221_. The Δ*ctb-1* mitotype from G_221_ does not result in a similar drop in mtDNA quantity in an N2 nuclear (wildtype) genetic background. Second, additional mtDNA mutations in *nd5* did not contribute to a further increase in mtDNA quantity. If the intracellular frequency of deleterious *nd5* mutations increased by benefitting from homeostatic mechanisms that keep sufficient levels of wildtype mtDNA within cells, we might expect a further increase in mtDNA quantity associated with these secondary mutations. Although the lack of copy-number increase does not explicitly reject the importance of mtDNA copy-number control in the proliferation of *nd5* mutations, it is inconsistent with the replicative advantage of smaller genomes.

In addition to the two hypotheses for selfish proliferation of mtDNA mutations discussed above (shorter genomes and copy-number), mitochondrial quality control mechanisms such as fusion-fission cycles and mitochondrial turnover (for example, mitophagy) have been shown to influence the fate of detrimental mtDNA mutations (Tam et al. 2013; Gitschlag et al. 2016; Meshnik et al. 2022). For instance, faster fusion-fission cycles can promote the proliferation of mutant mtDNA if mitochondria with high proportion of a mutation are not efficiently removed and can fuse with other mitochondria (Tam et al. 2015). If detrimental secondary mutations in mtDNA induce changes in mitochondrial dynamics such as fusion-fission, they may enhance the selfish properties of the original mutation.

Hypothetically, mtDNA mutations could be under directional selection, balancing selection or frequency-dependent selection (Gitschlag et al. 2016, 2020). In the case of balancing selection, one could imagine a scenario where two mitotypes are maintained in some optimal frequency based on complementation. This scenario may occur, for instance, if the *nd5* mutations in some way compensated for the negative effects of the deletion but happened to originate on a mtDNA molecule that did not contain the deletion. We lack evidence for this scenario. Selfish drive of these loss-of-function mutations (*ctb-1* deletion and the *nd5* frameshift) is expected to be countered by negative frequency-dependent selection. Since the *ctb-1* deletion and the *nd5* frameshift mutations affect essential mitochondrial genes, they cannot reach fixation. The negative frequency-dependent selection against the *ctb-1* deletion and *nd5* frameshift mutations could be due only to severe loss of individual fitness (for example, mortality or sterility) when the intra-individual frequency of these mutations exceeds some critical threshold. Additionally, within-individual processes such as apoptosis and mitophagy could also contribute. The combined frequency of the *nd5* T_8_ → T_9_ and T_9_ → T_10_ frameshift mutations and separately, the frequency of the *nd5* base substitution, can reach similar intra-individual frequencies as the *ctb-1* deletion. Hence, there is no evidence to suggest that these *nd5* mutations are under some additional negative frequency-dependent selection beyond what is already influencing the dynamics of the *ctb-1* deletion. The replay experiments are only testing for directional change in frequency under conditions where the frequency of these mutations is below the frequencies that we have already observed in the original MA experiment.

Finally, the location of the *nd5* gene upstream of the non-coding region suspected of containing a replication origin may play a role in enhancing the replication of the Δ*ctb-1* mitotype. It has been hypothesized that transcription plays a role in the proliferation of pathogenic mtDNA mutations, either directly such that increased transcription results in increased replication, or via a conflict between transcription and replication (Kowald and Kirkwood 2014; Røyrvik and Johnston 2020). The transcriptional consequences of *nd5* mutations could then influence the initiation of replication downstream. It is interesting that during the MA experiment that was the source of the Δ*ctb-1* mitotype, half of the mutations observed under extreme relaxation of selection (single individual bottlenecks) were located in *nd5* (Konrad et al. 2017). The majority (9/12) of these *nd5* mutations were frameshifts in one of two homopolymeric runs, one of which coincides with a mtDNA tRNA deletion in high frequency (Konrad et al. 2017). In light of the results in this paper that mutations in *nd5* may enhance the selfish properties of a *ctb-1* deletion, it is possible that some *nd5* mutations can have selfish properties on their own and can therefore be overrepresented in MA experiments where selection against individuals with poorly functioning mitochondria is severely limited. Furthermore, selfish mtDNA deletions described in a congeneric species, *C. briggsae*, are a consequence of recombination between *nd5* and an *nd5* pseudogene which is a partial duplicate copy of *nd5* (Howe and Denver 2008; Raboin et al. 2010; Phillips et al. 2015). The *nd5* pseudogene is widespread in natural populations of *C. briggsae* (Howe and Denver 2008). The presence of the *nd5* pseudogene increases the mutation load in *C. briggsae* mtDNA and it has been proposed that mutations in the *nd5* pseudogene are compensatory as they reduce the deletion frequency between the pseudogene and the intact *nd*5. However, why the pseudogene is tolerated despite obvious risks awaits an explanation. Furthermore, *Caenorhabditis* species *C. briggsae* and *C. sinica*, have acquired a second pseudo-*nd5* located in a tRNA cluster between *nd2* and *ctb-1* (Li et al. 2018). Novel mtDNA non-coding regions have arisen several times in the evolutionary history of *Caenorhabditis*, but *nd5* is the only genic source of these non-coding regions (Li et al. 2018).

The maintenance of optimal mitochondrial function within cells can be compromised by the accumulation of deleterious mtDNA mutations. Due to the large number of mtDNA copies per cell, selection against deleterious mutations is weak when they are rare. Furthermore, some mtDNA mutations can proliferate and increase in frequency within cells, in part by benefitting from services provided by wildtype mtDNA, and in part from compensatory mechanisms regulated by the nuclear genome. This situation, where mutant mtDNA can benefit from reduced functionality, has been referred to as the “tragedy of the cytoplasmic commons”, and if left unchecked, will lead to erosion of the genetic quality of mitochondria and a genetic conflict between the nuclear and mitochondrial genomes (Haig 2016). Many features of mitochondrial biology may have evolved to limit the risk of nuclear-mitochondrial genetic conflict, for example by reducing the opportunity for genetic mtDNA variation within cells. In plants and certain basal animal lineages, the mtDNA mutation rate is reduced by orders of magnitude relative to the nuclear genome (Palmer and Hebron 1988; Shearer et al. 2002). In animals, small mtDNA genome size, uniparental inheritance, reduction in the number of mtDNA copies during oogenesis, and mitochondrial quality control mechanisms that include mitophagy and apoptosis perform important roles in eliminating mutant mtDNA (Haig 2016; Havird et al. 2019). Some deleterious mtDNA variants still manage to escape the sophisticated mechanisms that have evolved to keep them in check, causing disease and contributing to mutational load. *C. elegans* has emerged as an excellent model to study mitochondrial biology including the proliferation of mtDNA deletions with implications for aging, disease and evolution (Estes et al. 2023; Onraet and Zuryn 2024). We show that mtDNA base substitutions can enhance the replicative advantage of an mtDNA deletion by unknown mechanisms. Because mtDNA exists as a nested hierarchy of populations—within mitochondria, within cells, within individuals and within populations of individuals—the study of the molecular mechanisms that influence the dynamics and fate of mtDNA mutations within cells fills in an important gap in the interphase between intra-individual and inter-individual selection. Further studies on how selfish properties of different kinds of mtDNA mutations arise are important to understand the mechanisms that are meant to keep them in check and under what conditions these mechanisms can be circumvented or exploited by rogue mtDNA molecules.

### Supplementary information

Supplementary Information “Dissecting the Sequential Evolution of a Selfish Mitochondrial Genome in *Caenorhabditis elegans*.”

## Data Availability

The phenotypic (fitness), heteroplasmic variant frequency, and mtDNA copy-number data supporting the findings of this study are available from the Dryad repository at the following link: 10.5061/dryad.1vhhmgr2z.
