# Supplementary material for: Dissecting the sequential evolution of a selfish mitochondrial genome in Caenorhabditis elegans
Source: Heredity (Edinb). 2024 Jul 5;133(3):186–97. doi: 10.1038/s41437-024-00704-2 (PMC11349875; doi:10.1038/s41437-024-00704-2)
Supplement: Supplementary file 1 — Supplementary Information “Dissecting the Sequential Evolution of a Selfish Mitochondrial Genome in Caenorhabditis elegans.” [file 41437_2024_704_MOESM1_ESM.pdf]

Supplementary Information

"Dissecting the Sequential Evolution of a Selfish Mitochondrial Genome in Caenorhabditis elegans."

Table S1 Frequency of each mtDNA mutation comprising the Δ*ctb-1* mitotype at MA generations G_0, G_{71}, G_{96}, G_{135}, G_{300}, and G_{350} following 17 generations of backcrossing. A horizontal bar "-" represents the absence of a mutation in a particular MA generation. "Present" denotes instances where the Δ*ctb-1* deletion was only confirmed by PCR and not by ddPCR.

MA Generation	Δ*ctb-1*	*nd5 frameshift* *(T_8→T_9)*	*nd5* *C→T substitution*	*nd5 frameshift* *(T_9→T_{10})*
G_0 (ancestral control)	-	-	-	-
G_{71}.A	0.79	-	-	-
G_{71}.B	0.81	-	-	-
G_{71}.C	0.68	-	-	-
G_{71}.D	0.68	-	-	-
G_{71}.E	0.65	-	-	-
G_{96}.A	0.83	0.06	-	-
G_{96}.B	0.83	0.06	-	-
G_{96}.C	0.93	0.16	-	-
G_{96}.D	0.91	0.30	-	-
G_{96}.E	0.87	0.14	-	-
G_{135}.A	present	0.77	0.88	-
G_{135}.B	present	0.77	0.87	-
G_{135}.C	present	0.66	0.81	-
G_{135}.D	present	0.61	0.64	-
G_{300}.B	present	0.35	0.96	0.59
G_{300}.C	present	0.40	0.92	0.52
G_{300}.E	present	0.45	0.95	0.49
G_{350}..C	0.89	0.23	0.87	0.67
G_{350}.N	0.82	0.13	0.83	0.73
G_{350}.T	0.85	0.21	0.91	0.71
G_{350}.U	0.86	0.21	0.83	0.68

Table S2 Two-level nested ANOVA for relative mean productivity, developmental rate, longevity, and survivorship to adulthood of the ancestral control (G_0) and Δ*ctb-1* mitotypes of MA line 1G from differing time-points and different combinations of mtDNA mutations (generations G_{71}, G_{96}, G_{221}, and G_{350}) backcrossed into a common N2 nuclear background.

Source of variation	*df*	SS	MS	F_s	F_s'
Productivity					
Among groups (mitotypes)	4	18.067	4.517	51.92	55.12****
Among lines (replicates)	18	1.559	0.087	1.06 *ns*	
Within replicates (error)	307	25.157	0.082		
Total	329	44.724			
Developmental Rate					
Among groups (mitotypes)	4	0.720	0.180	10.43	19.42****
Among lines (replicates)	18	0.311	0.017	1.86 *ns*	
Within replicates (error)	306	2.837	0.009		
Total	328	3.866			
Longevity					
Among groups (mitotypes)	4	1.447	0.362	1.82	2.65*
Among lines (replicates)	18	3.585	0.199	1.46 *ns*	
Within replicates (error)	302	41.216	0.136		
Total	324	46.272			
Survivorship to Adulthood					
Among groups (mitotypes)	4	1.328	0.332	4.67	17.04****
Among lines (replicates)	18	1.281	0.071	3.65****	
Within replicates (error)	313	6.099	0.019		
Total	335	8.787			

* significance level of 0.05
** significance level of 0.01
*** significance level of 0.001
**** significance level of 0.0001
ns, nonsignificant

Table S3 Test of pair-wise differences in four fitness-related traits (productivity, developmental rate, longevity, and survivorship to adulthood) between lines with mitotypes from different stages of the MA experiment (ancestral control G_0, G_{71}, G_{96}, G_{221}, G_{350}) using the Tukey–Kramer HSD method of multiple comparisons among pairs of means.

Productivity

	G_0	G_{71}	G_{96}	G_{221}	G_{350}
G_0	-	0.144	0.138	0.148	0.132
G_{71}	0.207*	-	0.139	0.149	0.133
G_{96}	0.315*	0.108	-	0.143	0.126
G_{221}	0.547*	0.340*	0.232*	-	0.137
G_{350}	0.641*	0.433*	0.325*	0.093	-

Developmental Rate

	G_0	G_{71}	G_{96}	G_{221}	G_{350}
G_0	-	0.050	0.048	0.051	0.045
G_{71}	0.008	-	0.048	0.051	0.046
G_{96}	0.043	0.036	-	0.049	0.043
G_{221}	0.089*	0.081*	0.045	-	0.047
G_{350}	0.117*	0.109*	0.074*	0.029	-

Longevity

	G_0	G_{71}	G_{96}	G_{221}	G_{350}
G_0	-	0.187	0.182	0.172	0.198
G_{71}	0.058	-	0.182	0.172	0.198
G_{96}	0.022	0.036	-	0.166	0.192
G_{221}	0.157	0.099	0.135	-	0.183
G_{350}	0.160	0.102	0.138	0.003	-

Survivorship to Adulthood

	G_0	G_{71}	G_{96}	G_{221}	G_{350}
G_0	-	0.076	0.073	0.077	0.07
G_{71}	0.066	-	0.071	0.075	0.068
G_{96}	0.142*	0.076*	-	0.072	0.064
G_{221}	0.200*	0.134*	0.058	-	0.069
G_{350}	0.088*	0.022	0.054	0.112*	-

Absolute differences among all pairs of trait means *i* and *j* are listed below the diagonal and their critical MSD*ij* values above the diagonal. A pair of means is declared significantly different at $\alpha = 0.05$ if their absolute difference equals or exceeds their MSD values (indicated by an asterisk).

* A pair of means that are significantly different at an experiment-wise error rate of $\alpha = 0.05$.

A

```
wt ctb-1    MKINNSLLNF VNGMLVTLPS SKTLTLSWNF GSMLGMVLIF QILTGTFLAF
Δctb-1      MKINNSLLNF VNGMLVTLPS SKTLTLSWNF GSMLGMVLIF QILTGTFLAF

wt ctb-1    YYTPDSLMAF STVQYIMYEV NFGWVFRIFH FNGASLFFIF LYLHIFKGLF
Δctb-1      YYTPDSLMAF STVQYIMYEV NFGWVFRIFH FNGASLFFIF LYLHIFKGLF

wt ctb-1    FMSYRLKKVW MSGLTIYLLV MMEAFMGYVL VWAQMSFWAA VVITSLLSVI
Δctb-1      FFIIFYLLMQ FCVLFQMKS- ---------- ---------- ----------

wt ctb-1    PIWGPTIVTW IWSGFGVTGA TLKFFFVLHF LLPWAILVIV LGHLIFLHST
Δctb-1      ---------- ---------- ---------- ---------- ----------

wt ctb-1    GSTSSLYCHG DYDKVCFSPE YLGKDAYNIV IWLLFIVLSL IYPFNLGDAE
Δctb-1      ---------- ---------- ---------- ---------- ----------

wt ctb-1    MFIEADPMMS PVHIVPEWYF LFAYAILRAI PNKVLGVIAL LMSIVTFYFF
Δctb-1      ---------- ---------- ---------- ---------- ----------

wt ctb-1    ALVNNYTSCL TKLNKFLVFM FIISSTILSW LGQCTVEDPF TILSPLF
Δctb-1      ---------- ---------- ---------- ---------- -------
```

B

```
wt nd5        MNISIFLIGF VFFMGGISVW LMPTFKLGIF FLEWDFLSLK FNFYFNSILF
nd5 nonsyn    MNISIFLIGF VFFMGGISVW LMPTFKLGIF FLEWDFLSLK FNFYFNSILF
nd5 (+1)      MNISIFLIGF VFFMGGISVW LMPTFKLGIF FFSMSFFKVK ----------
nd5 (+2)      MNISIFLIGF VFFMGGISVW LMPTFKLGIF FF-------- ----------

wt nd5        SFILFLVTFS VLVFSTYYLN SELNFNYYYF VLLIFVGSMF SLNFSNSIFT
nd5 nonsyn    SFILFLVTFS VLVFSTYYLN SELNFNYYYF VLLIFVGSMF SLNFSNSIFT
nd5 (+1)      ---------- ---------- ---------- ---------- ----------
nd5 (+2)      ---------- ---------- ---------- ---------- ----------

wt nd5        MLLSWDLLGI SSFFLVLFYN NWDSCSGAMN TALTNRLGDY FMFVFFGLSV
nd5 nonsyn    MLLSWDLLGI SSFFLVLFYN NWDSCSGAMN TALTNRLGDY FMFVFFGLSV
nd5 (+1)      ---------- ---------- ---------- ---------- ----------
nd5 (+2)      ---------- ---------- ---------- ---------- ----------

wt nd5        FSGYYFLSFS MFSSYMSLLL LLTAFTKSAQ FPFSSWLPKA MSAPTPVSSL
nd5 nonsyn    FSGYYFLSFS MFSSYMSLLL LLTAFTKSAQ FPFSSWLPKA MSAPTPVSSL
nd5 (+1)      ---------- ---------- ---------- ---------- ----------
nd5 (+2)      ---------- ---------- ---------- ---------- ----------

wt nd5        VHSSTLVTAG LILLMNFNNL VMQKDFISFV LIIGLFTMFF SSLASLVEED
nd5 nonsyn    VHSSILVTAG LILLMNFNNL VMQKDFISFV LIIGLFTMFF SSLASLVEED
nd5 (+1)      ---------- ---------- ---------- ---------- ----------
nd5 (+2)      ---------- ---------- ---------- ---------- ----------

wt nd5        LKKVVALSTL SQMGFSMVTL GLGLSFISFI HLVSHALFKS CLFMQVGYII
nd5 nonsyn    LKKVVALSTL SQMGFSMVTL GLGLSFISFI HLVSHALFKS CLFMQVGYII
nd5 (+1)      ---------- ---------- ---------- ---------- ----------
nd5 (+2)      ---------- ---------- ---------- ---------- ----------

wt nd5        HCSFGQQDGR NYSNNGNLPN FIQLQMLVTL FCLCGLIFSS GAVSKDFILE
nd5 nonsyn    HCSFGQQDGR NYSNNGNLPN FIQLQMLVTL FCLCGLIFSS GAVSKDFILE
nd5 (+1)      ---------- ---------- ---------- ---------- ----------
nd5 (+2)      ---------- ---------- ---------- ---------- ----------

wt nd5        LFFSNNYMMF FSLMFFVSVF LTFGYSFRLW KSFFLSFNKV MNHYSSTVFM
nd5 nonsyn    LFFSNNYMMF FSLMFFVSVF LTFGYSFRLW KSFFLSFNKV MNHYSSTVFM
nd5 (+1)      ---------- ---------- ---------- ---------- ----------
nd5 (+2)      ---------- ---------- ---------- ---------- ----------

wt nd5        NFLSLVLVIF SISFLWWMNF NLLNIPSLFL YVDFFGPLVF LFMMIFLSFL
nd5 nonsyn    NFLSLVLVIF SISFLWWMNF NLLNIPSLFL YVDFFGPLVF LFMMIFLSFL
nd5 (+1)      ---------- ---------- ---------- ---------- ----------
nd5 (+2)      ---------- ---------- ---------- ---------- ----------

wt nd5        ILKMLFKELM YKFLVDYLAK NSIYKMKNLK FMDLFLNNIN SKGYTLFLSS
nd5 nonsyn    ILKMLFKELM YKFLVDYLAK NSIYKMKNLK FMDLFLNNIN SKGYTLFLSS
nd5 (+1)      ---------- ---------- ---------- ---------- ----------
nd5 (+2)      ---------- ---------- ---------- ---------- ----------

wt nd5        GMFKNYYLKS LNFNSVVVLI FIFFMIC
nd5 nonsyn    GMFKNYYLKS LNFNSVVVLI FIFFMIC
nd5 (+1)      ---------- ---------- -------
nd5 (+2)      ---------- ---------- -------
```

Fig. S1: Protein alignments of the wildtype mitochondrial **A** *ctb-1* and **B** *nd5* genes in *C. elegans* and four spontaneously arising mutations (one large deletion, small insertions and one nonsynonymous mutation) during mutation accumulation in line 1G.
